# Hunting for Familial Parkinson’s Disease Mutations in the Post Genome Era

**DOI:** 10.3390/genes12030430

**Published:** 2021-03-17

**Authors:** Steven R. Bentley, Ilaria Guella, Holly E. Sherman, Hannah M. Neuendorf, Alex M. Sykes, Javed Y. Fowdar, Peter A. Silburn, Stephen A. Wood, Matthew J. Farrer, George D. Mellick

**Affiliations:** 1 Griffith Institute for Drug Discovery, Griffith University, Don Young Road, Nathan, QLD 4111, Australia; hannah.neuendorf@qimrberghofer.edu.au (H.M.N.); a.sykes@griffith.edu.au (A.M.S.); j.fowdar@griffith.edu.au (J.Y.F.); s.wood@griffith.edu.au (S.A.W.); g.mellick@griffith.edu.au (G.D.M.); 2 Djavad Mowafhagian Centre for Brain, University of British Columbia, Vancouver, BC V6T 1Z3, Canada; iguella@can.ubc.ca (I.G.); hsherman@can.ubc.ca (H.E.S.); mfarrer@can.ubc.ca (M.J.F.); 3 Centre for Clinical Research, University of Queensland, Brisbane, QLD 4029, Australia; p.silburn@uq.edu.au

**Keywords:** familial, multi-incident, parkinsonism, movement disorder, exome, mutation

## Abstract

Parkinson’s disease (PD) is typically sporadic; however, multi-incident families provide a powerful platform to discover novel genetic forms of disease. Their identification supports deciphering molecular processes leading to disease and may inform of new therapeutic targets. The *LRRK2* p.G2019S mutation causes PD in 42.5–68% of carriers by the age of 80 years. We hypothesise similarly intermediately penetrant mutations may present in multi-incident families with a generally strong family history of disease. We have analysed six multiplex families for missense variants using whole exome sequencing to find 32 rare heterozygous mutations shared amongst affected members. Included in these mutations was the *KCNJ15* p.R28C variant, identified in five affected members of the same family, two elderly unaffected members of the same family, and two unrelated PD cases. Additionally, the *SIPA1L1* p.R236Q variant was identified in three related affected members and an unrelated familial case. While the evidence presented here is not sufficient to assign causality to these rare variants, it does provide novel candidates for hypothesis testing in other modestly sized families with a strong family history. Future analysis will include characterisation of functional consequences and assessment of carriers in other familial cases.

## 1. Introduction

Parkinson’s disease (PD) typically presents sporadically; however, 15.7% of cases report an affected first-degree relative [1]. Further, descriptions of familial clusters have been recorded since the late 19th century [2]. It was not until the late 20th century that the first genetic mutation, *SNCA* p.A53T, was found to segregate with disease in a large multi-incident family from Contursi, Italy [3]. In following years, additional mutations sufficient to cause late-onset PD were identified in other familial clusters, including *SNCA* p.A30P [4], *LRRK2* p.R1441G, p.Y1699C [5], p.R1441C [6], and *VPS35* p.D620N [7,8]. While notably incomplete, these mutations possess generally high age-dependent penetrance with more than 95% of carriers developing disease by 80 years [9]. The penetrance of the *LRRK2* p.G2019S mutation in carriers from European decent is markedly lower: 42.5–68% at 80 years depending on the method used [9,10]. Consistently, this mutation can be found in patients without a family history of disease [11].

The advancing genomic sequencing platforms have supported the hunt for novel disease-causing mutations, including the *VPS35* p.D620N variant [7,8]. More recently, extensive genetic analysis of other large multi-incident families suggested putative roles for *DNAJC13* p.N855S [12] and *TMEM230* p.R141L [13] (same family), *CHCHD2* p.T61I [14] and *PLXNA4* p.S657N [15], among others, in late-onset PD. While assessment of the pathogenicity of these variants is on-going and complicated by their rarity, identification of novel disease-causing mutations provides opportunities for molecular therapies and biomarker discovery. However, although large multigenerational multi-incident families provide the greatest power to discover mutations, these families are uncommon and were often the focus of early linkage analyses [16,17,18]. Further, if a genetic cause is determined in these kindreds, the mutation would likely carry high penetrance due to the nature of the family presentation.

We had previously reported 137 multi-incident families in Queensland [1], which after extensive genetic analysis of 18 probands, we had only identified one kindred ascribing to a known genetic form of disease. These families presented with a strong family history but did not conform strictly to a dominant or recessive inheritance. We hypothesise that, similar to the *LRRK2* p.G2019S mutation, other genetic changes possess a moderate to strong effect size presenting as intermediate penetrance. Consequently, kindreds with these mutations would not present classical autosomal dominance, but rather would have a sparse but generally strong family history of disease.

To this end, we aimed to identify putative mutations that confer intermediate penetrance using six moderately sized multi-incident kindreds. We provide preliminary evidence of 32 rare heterozygous missense genetic variants, which we hypothesise may have a role in disease for these families. Through reporting these putative disease-causing variants, we aim to support the search of intermediately penetrant mutations leading to dominant late-onset parkinsonism.

## 2. Materials and Methods

### 2.1. Patient Recruitment

As previously described, families reporting more than three family members with parkinsonism (deemed multi-incident) were selected from the Queensland Parkinson’s Project [1]. Families with at least affected cousin-pairs were selected for the current variant discovery analysis. Incomplete penetrance was notable in all families; however, this was expected for intermediately penetrant sequence variants. Patients were diagnosed by a movement disorders specialist using established criteria [19]. Patients were described to have typical PD, with an average age at onset of 64.0 years (+/− 10.4 y), ranging from 48–87 years. It is noteworthy that one patient from family #002 was considered asymptomatic as positron emission tomography imaging of ^11^C-d-*threo*-methylphenidate found significantly lower levels in the left-caudate when compared to age-matched controls. Leukocyte DNA was extracted from the proband, available affected family members, and, when available, elderly unaffected members of the family. The probands were then screened absent for known genetic forms of disease, including single nucleotide variations, small insertion and deletions, expansions of repetitive regions in *ATXN2*, *ATXN3* and *TBP*, and copy number variations in *SNCA* and *PARK2* [1]. 

### 2.2. Whole Exome Sequencing

Similarly, as described previously, patient DNA underwent whole exome sequencing using the Nextera Rapid Capture Exome Enrichment capture library and sequencing on the Illumina MiSeq (Illumina, San Diego, CA, USA) at the Griffith University DNA Sequencing Facility, prepared using the Ion AmpliSeq capture kit and sequenced using the Ion Torrent (Thermo Fisher Scientific, Waltham, MA, USA) or sent to PerkinElmer Genomics for sequencing on the Illumina HiSeq [1] (Supplementary Table S1). Joint genotyping variant calling was conducted using the HaplotypeCaller from the GenomeAnalysis ToolKit (v3.5) for the MiSeq data, while the Torrent Suite (v4.0) was used for Ion Torrent data. Separate variant caller files were parsed and combined using in-house scripts, leveraging GATK, SAMtools, and bedtools2. These scripts combined variant on the basis of chromosome, position, reference, and alternative alleles for each family individually. While similar genotype was not required for the merge, variants with low depth (≤2 reads) were flagged and later omitted. Variants were annotated using the ANNOVAR package [20] with the general frequency from the Genome Aggregation Database (gnomAD, v2) [21].

### 2.3. Variant Segregation Analysis

Variants were selected from each kindred based on the following criteria in BASH scripts: (1) present in affected members of the family while taking into consideration incomplete penetrance, (2) had a minor allele frequency of <0.0001 in the gnomAD, (3) were exonic or splicing region (RefSeq v61), (4) were a missense allele. Further, likely sequence artefacts were removed by omitting variants that were also seen in >30% of the MiSeq in-house datasets (2n = 48) or >0.5% of the AnnEx Annotated Exomes browser (2n = 5902, https://annex.can.ubc.ca, accessed on 4 December 2020) for Ion Torrent data. Variants identified within a kindred on the same sequencing platform were confirmed using Sanger sequencing on the genetic analyzer 3131xl (Applied Biosystems, Foster City, CA, USA) at the Griffith University DNA Sequencing Facility (Supplementary Table S2). Variants identified in the same family across multiple sequencing platforms did not undergo Sanger confirmation as the likelihood of common sequence artefacts are rare. When DNA samples were unavailable for parents, inference of carrier status was reasoned by the rarity of the variant and the genetic relatedness of descendent cousin-pairs undergoing whole exome sequencing, suggesting inheritance by descent as opposed to coincidental de novo occurrences.

### 2.4. Mutation Frequency

Targeted genotyping in other PD cases were conducted for *KCNJ15* p.R28C, *SON* p.S1595P and *PASK* p.P519L (family #002), *ARL14EP* p.A146V (family #431), and *SIPA1L1* p.R236Q (family #433). Due to differences in data availability from whole exome sequencing, the genotyping assays were conducted on different samples and at different times. These variants were chosen based on minor allele frequency or large family size. The Sequenom MassARRAY (Agena Bioscience, San Diego, CA, USA) platform was used to screen *KCNJ15* p.R28C, *SON* p.S1595P, and *PASK* p.P519L in 7018 PD alleles and 7224 alleles from non-PD subjects from the Genetic Epidemiology of Parkinson’s Disease (GeoPD) cohort, encompassing seven countries. Due to the rarity of the variants, no comparisons were made between ethnicities. Further, a TaqMan assay (Thermo Fisher Scientific, Waltham, MA, USA) screened the *KCNJ15* p.R28C variant in an additional 3230 sporadic PD alleles, 1632 familial PD alleles, and 2788 alleles from non-PD subjects (Supplementary Table S3). While non-PD subjects were not collected from an alternative disease sample collection, each subject’s medical history was not assessed in detail and may be affected by other diseases. High resolution melt analysis method then screened the *ARL14EP* p.A146V and *SIPA1L1* p.R236Q variants in 102 alleles from PD cases from multi-incident families, 282 alleles from familial PD cases, and 330 alleles from sporadic PD cases. Additionally, all variants were assessed on the AnnEx browser (2n = 5902, PD 2n =1048), PD DNA variant browser (https://pdgenetics.shinyapps.io/VariantBrowser/, accessed on 19 February 2021, total 2n = 204,254, PD 2n = 56,906) [22], and the Medical Genome Reference Bank (MGRB, 2n = 5690) [23]. The PD DNA variant browser contained data from the United Kingdom BioBank (UKB, unaffected 2n = 76,526) [24]. 

## 3. Results

**Figure 1:**
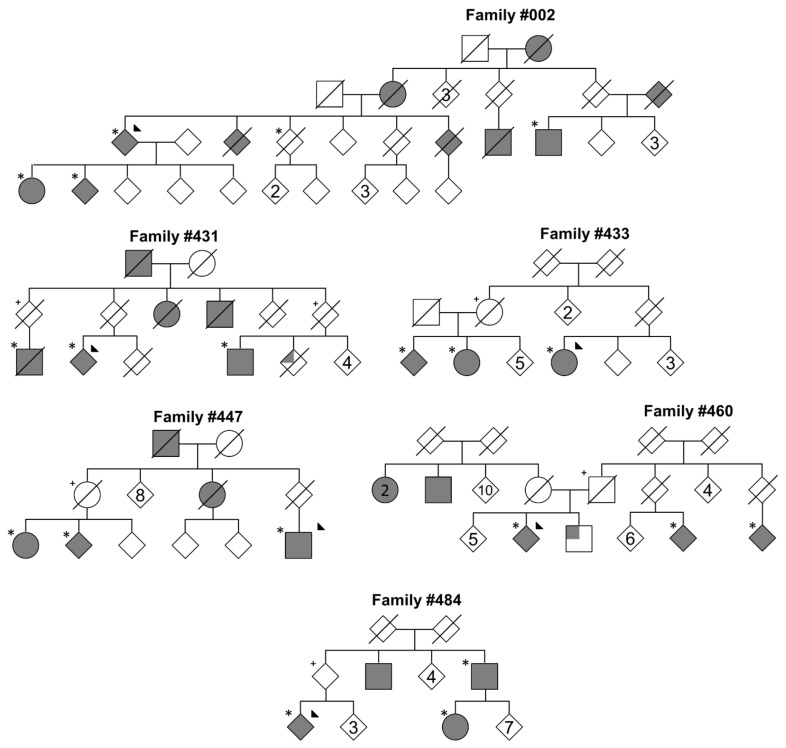
Six multi-incident families from the Queensland Parkinson’s Project. Full shaded shapes represent Parkinson’s disease (PD). Upper shaded quadrants represent an undiagnosed tremor. Those with a star indicate Whole Exome Sequencing (WES) sequencing. Figures depicted with plus sign represent individuals that were both >80 years of age and inferred carrier of variants. Triangle suggests proband case. Gender disclosed and minimum data shown to protect the privacy of the participants.

Probands from six modestly sized multi-incident families from the Queensland Parkinson’s Project were assessed for disease-causing point mutations, indels, trinucleotide expansions, and gene dosage in *SNCA* and *PARK2* [1]. Subsequently, affected family members underwent whole exome sequencing and variant segregation analysis (Figure 1), identifying a total of 32 rare putative disease-causing coding variants within the families (Table 1). All candidate variants were single nucleotide variations, and with the exception of *UNC13B* c.3188+1G>A, resided within exon boundaries. Three families were found to have fewer than three candidate-variants shared between affected members: family #002, #431, and #433. It is noteworthy that incomplete penetrance to the age of 80 years was observed in all families. 

A survey of these variants was performed in the AnnEx browser, identifying a number of non-PD carriers, who were reported to have schizophrenia, multiple sclerosis, or was not disclosed. The exceptions were *FAM71B* p.I318T, which was identified in a patient with frontotemporal dementia, and *ZNF75A* p.Q212E, which was identified in two of three members of another multi-incident family from the Queensland Parkinson’s Project, family #006 (Supplementary Figure S1). Using the same methodology as described in this report, the #006 kindred had 20 unique candidate variants that were shared across all three affected members. Additionally, a survey of the PD genetics found several other PD cases with the same mutations; however, *TAF1C* p.R346Q and *UNC13B* c.3188+1G>A were only found in PD cases (Table 1). The combined UKB controls and MGRB, 2n = 82,216 suggest these variants are rare in those datasets as well. 

Due to the number of affected members in family #002 (Figure 1), all three candidate variants were selected for targeted genotyping in unrelated PD cases. This analysis initially identified the *KCNJ15* p.R28C variant in an isolated Italian familial case with an age at onset of 63 years, while the *PASK* p.519L and *SON* p.S1595P were absent in 6534 and 5748 PD alleles, respectively. The *KCNJ15* p.R28C was then assessed in an additional 4862 PD alleles, identifying another sporadic PD carrier from Australia, age at onset of 51 years. Interestingly, the *SON* and *KCNJ15* variants reside within 4.7 Mb on chromosome 21; however, the *SON* variant was not present in the sporadic Australian or the familial Italian patients. Targeted genotyping did not find the *KCNJ15* and *SON* variants in non-PD alleles, while the *PASK* p.519L substitution was identified in four alleles from non-PD subjects. Three additional members above the age of 80 years from the #002 kindred were assessed for the variants, which identified the *KCNJ15* gene mutation in two subjects, *SON* in two, and the *PASK* variant in three subjects. Collectively, the *KCNJ15* p.R28C mutation was identified in five PD cases and two unaffected members above the age of 80 years from family #002 (Supplementary Figure S2 Panel A), and two unrelated PD cases without strong family history of disease.

Kindred #433 presented with one candidate mutation, *SIPA1L1* p.R236Q. This variant was absent in one elderly sibling (>80 years) and through inference, present in an elderly parent before death (Figure 1 and Supplementary Figure S2 Panel B). This mutation was selected for targeted genotyping in 714 PD alleles, which identified one additional familial case from Australia, age at onset of 60 years. While kindred #431 presented with three candidate variants, *ARL14EP* p.A146V was a novel variant, and subsequently genotyped in unrelated PD alleles. This analysis did not find additional carriers of the mutation.

## 4. Discussion

Here, we aim to address the occurrence of rare variants that can contribute to disease with an intermediate penetrance, as seen in the *LRRK2* p.G2019S mutation [9,10]. The discovery of such variants may provide further insights to disease in PD families, as well as potential new molecular targets for therapies and biomarker development. Hence, we utilised whole exome sequencing in a kindred-based model to identify rare sequence variants shared between affected members of each family, and then assessed variant frequency in other cases and databases disclosing phenotype. This analysis identified *SIPA1L1* p.R236Q and *KCNJ15* p.R28C as two putative candidates for disease. The *SIPA1L1* gene encodes the SPAR1 protein, which has a role in actin organisation of dendritic spines [25] and neuron plasticity [26]. *KNCJ15* encodes Kir4.2, an inwardly rectifying potassium channel. A knockdown model of this protein was found to reduce the ability of epithelial cells to sense electrical fields, leading to compromised electrotaxis [27]. The Kir4.2 is moderately expressed in glial cells from the caudate [28], and potassium channels have been noted in the activation of microglial [29,30]. However, we are cautious to speculate further on functional consequences of these mutations, as although we list 32 rare variants that are shared between affected members of the family, there are a number of notable assumptions, challenges, and limitations of this study. 

Firstly, we anticipate at least 14.4% of monogenic families contain a phenocopy [31]. Due to the relatively small sample size of each kindred analysed, the presence of such a case would lead to an erroneous variant list. We have characterised six families to reduce this risk; however, one such family at risk was kindred #002, as one affected member was identified as prodromal due to reductions in caudate ^11^C-d-*threo*-methylphenidate binding. While this method was shown effective in asymptomatic *LRRK2* p.R1441C carriers [32], the application to this family member is unknown. Secondly, although advancements in DNA sequencing technology have improved accessibility of large-scale genome data, a substantial number of analyses and variant datasets are still restricted to coding and splice variants. For instance, we estimated one of the exome capture methods we used, Nextera rapid capture kit, only covered 0.22% of large intergenic non-coding RNA [33]; thus, our and other exome-based analyses under-represent variants in these areas. As genome data becomes more accessible, we anticipate population frequency of intergenic variants will become more refined and improve the value of a whole genome sequencing approach to variant discovery.

Thirdly, while assessment of current genetic databases, such as gnomAD, Medical Genome Reference Bank (MGRB), and United Kingdom BioBank (UKB), provide good estimations of the frequency of variants in particular study populations, there are a few notable limitations. The age at recruitment of participants is not exclusively above the age of 80 years; for instance, in gnomAD v2 only 1935 of the 125,748 were above 80 years [21]. This increases the likelihood of identifying a variant intermediately contributing to disease in an unaffected subject, as they could be pre-symptomatic. Additionally, due to the heterogenous study designs contributing to the gnomAD, the participant’s status of PD or family history of PD is not clearly defined. Thus, we cannot determine if participants from gnomAD carrying the rare variants identified in this study would develop PD.

While the MGRB and UKB projects collect more phenotypic information about participants, these too are subject to similar limitations. The UKB describes only 10% of participants are over 80 years (data-field 34); while 3602 participants had PD (data-field 131022), 19,460 parents of participants were also reported to have PD (data-fields 20107 and 20110) [24,34]. In addition, the UKB dataset had identified 30.3% of participants were related up to the third-degree [34], indicating frequency of rare variants may be exacerbated by these familial clusters. Thus, while *KCNJ15* p.R28C and *SIPA1L1* p.R236Q were identified in participants of gnomAD, UKB, and MGRB, the disease outcomes and family histories of these subjects are unclear and warrant further investigation. Similarly, while these sequence mutations were also carried by unrelated PD cases, this is not sufficient evidence to suggest causality as these carriers did not present with a strong family history of disease. A noteworthy cofounding factor, as documented in the *LRRK2* p.G2019S mutation, is varying penetrance in different populations, genetic architecture, and environmental stressors [9,35]. This further increases the challenge to determine if these variants are pathogenic or simply coincidental rare sequence mutations found in affected family members.

As large multi-incident families are uncommon, other models have been employed and previously aimed to discover putative disease-implicated variants, including analysis of isolated populations [36], population-specific burden analyses [37,38], burden pathway analyses [39,40], trio analyses [41], and an integrated genetic cohort and experimental model [42]. By presenting these putative variants, we aim to supplement these other approaches and to generate candidates for hypothesis testing. Similar to Jansen and colleagues [42] but beyond the scope of this manuscript, a number of our putative targets will undergo functional characterisation in cellular models to determine the effect of the amino acid changes. For instance, identifying the impact the *KCNJ15* p.R28C variant has on channel formation with and without the presence of endogenous potassium channel proteins.

## 5. Conclusions

The advancements in DNA-sequencing platforms has increased accessibility to human genome data. Leveraging these advancements, we address the occurrence of mutations that confer intermediate penetrance for disease. This analysis has identified 32 rare heterozygous coding genetic variants shared within affected members of six moderately sized multi-incident kindreds, including *KCNJ15* p.R28C and *SIPA1L1* p.R236Q which were identified in unrelated PD cases. While this analysis alone is under-powered to ascribe these mutations as pathogenic, it does provide candidates for hypothesis testing. Supported by the growing amount of genome data from PD patients, the information presented here may aid the global search for novel disease-causing mutations in other modestly sized late-onset PD families and lead to novel therapeutics.

## Figures and Tables

**Table 1:** Candidate Genetic Variants.

Family	# of Affected Carriers	# of Unaffected Carriers I	Gene	Transcript	Protein Change	GnomAD Exome MAF	AnnEx State (Count)	PD Genetics	UKB II and MGRB	CADD (Phred)
#002	5	2	*KCNJ15*	NM_001276436	p.R28C	1.22 × 10^−5^	Non-PD (1)	0	4	16.9
			*SON*	NM_138927	p.S1595P	4.06 × 10^−5^	0	0	13	2.864
		3	*PASK*	NM_001252120	p.P519L	2.03 × 10^−5^	0	2	3	11.58
#431	3	2	*ARL14EP*	NM_152316	p.A146V	0.00	0	0	0	21.5
			*HYDIN*	NM_001270974	p.A2271E	4.44 × 10^−6^	0	0	0	4.081
			*PTPRA*	NM_080840	p.R223W	5.32 × 10^−5^	Non-PD (2)	0	43	28.6
#433	3	1	*SIPA1L1*	NM_001284247	p.R236Q	2.85 × 10^−5^	0	0	13	16.02
#447	3	1	*ZNF462*	NM_021224	p.I1523V	4.06 × 10^−6^	0	0	0	11.9
			*DUSP19*	NM_001142314	p.I111R	4.06 × 10^−6^	0	0	0	24
			*KCTD1*	NM_001136205	p.G134R	4.07 × 10^−6^	0	0	0	4.792
			*TAF1C*	NM_001243158	p.R346Q	8.29 × 10^−6^	0	1	0	2.672
			*DARS2*	NM_018122	p.S59L	1.22 × 10^−5^	0	0	0	15.29
			*EXPH5*	NM_001144765	p.T920S	2.88 × 10^−5^	0	0	1	10.15
			*FAM71B*	NM_130899	p.I318T	6.10 × 10^−5^	Non-PD (2) III	1	2	0.01
			*CCDC180*	NM_020893	p.R1684C	8.53 × 10^−5^	0	0	3	12.13
#484	3	1	*TCP10L*	NM_144659	p.I54F	0.00	0	0	0	5.434
			*ADCY4*	NM_001198568	p.H760Q	4.10 × 10^−6^	0	0	6	11.05
			*USP42*	NM_032172	p.H952R	5.69 × 10^−6^	0	0	1	14.75
			*UNC13B*	NM_006377	c.3188+1G>A	1.63 × 10^−5^	0	1	0	25.6
			*CFTR*	NM_000492	p.K1080R	2.85 × 10^−5^	Non-PD (2)	0	1	28.7
			*NINL*	NM_025176	p.Q1232H	3.58 × 10^−5^	0	0	9	12.17
			*CST5*	NM_001900	p.S66N	3.67 × 10^−5^	Non-PD (3)	4	12	11.77
#460	3 IV	1	*SLC2A12*	NM_145176	p.S357L	0.00	0	0	0	22
			*DOCK3*	NM_004947	p.R392W	0.00	0	0	0	18.96
			*TPR*	NM_003292	p.K1038N	0.00	0	0	4	16.34
			*DNAH1*	NM_015512	p.K1792R	4.06 × 10^−6^	0	0	0	21.3
			*PCDHGA7*	NM_018920	p.S667G	1.63 × 10^−5^	Non-PD (1)	1	12	12.13
			*MYOT*	NM_001135940	p.N30K	2.03 × 10^−5^	Non-PD (1)	1	10	6.21
			*KIF9*	NM_022342	p.R287W	2.44 × 10^−5^	0	0	0	20.4
			*DNAJC12*	NM_021800	p.T99M	4.06 × 10^−5^	Non-PD (1)	2	6	14.86
			*SLAMF8*	NM_020125	p.V234E	7.31 × 10^−5^	Non-PD (1)	2	21	17.11
			ZNF75A	NM_153028	p.Q212E	8.95 × 10^−5^	Non-PD (1), PD(2) V	0	28	14.51

I Carriers >80 years, II UKB controls, III Frontotemporal Dementia, IV Undiagnosed tremor not included, V family #006. Minor Allele Frequency: MAF; Parkinson’s disease: PD; United Kingdom BioBank: UKB; Medical Genome Reference Bank: MGRB; Combined Annotation Dependent Depletion: CADD. (1)–(3): These are the counts of that state.

## Data Availability

Whole exome sequencing data presented here has been stored in the AnnEx “Annotated Exomes” browser (https://annex.can.ubc.ca, accessed on 16 March 2021) and can be acssed via this platform or through contacting the authors.

## Supplementary Materials

The following are available online at https://www.mdpi.com/2073-4425/12/3/430/s1, Figure S1. Queensland kindred, #006, with two af-fected members carrying the ZNF75A p.Q212E mutation, as indicated by the star. Whole exome sequencing performed on three affected siblings, finding 20 shared rare sequence variants. Full shaded shapes represent PD. Triangle suggests proband case. Gender disclosed and minimum data shown to protect the privacy of the participants. Figure S2. (A) Segregation of the KCNJ15 p.R28C variant in family #002. (B) Segregation of SIPA1L1 p.R236Q variant in family #433. Full shaded shapes represent PD. M represents heterozygous mutation carrier. Italicized M represents inferred mutation carrier >80 years. W represents confirmed homozygous reference >80 years. Triangle suggests proband case. Gender disclosed and minimum data shown to protect the privacy of the participants. Table S1. Details of affected family members and sequencing method. Table S2. Primer designs. Table S3. Genotyping variants from #002 by country.
