# Supplementary material for: Hunting for Familial Parkinson’s Disease Mutations in the Post Genome Era
Source: Genes (Basel). 2021 Mar 17;12(3):430. doi: 10.3390/genes12030430 (PMC8002626; doi:10.3390/genes12030430)
Supplement: Supplementary file 1 [file genes-12-00430-s001.pdf]

genes MDPI

Supplementary A

Table S1. Details of affected family members and sequencing method.

Family #	Age-at-onset	Sequencing Method
**002**	**63**	**Perkin Elmer HiSeq**
**002**	**53**	**Perkin Elmer HiSeq**
**002**	**66**	**Nextera MiSeq**
**002**	**40 ‡**	**Ampliseq Ion Torrent**
**431**	**ND**	**Perkin Elmer HiSeq**
**431**	**62**	**Perkin Elmer HiSeq**
**431**	**66**	**Ampliseq Ion Torrent**
**433**	**60**	**Ampliseq Ion Torrent**
**433**	**59**	**Ampliseq Ion Torrent**
**433**	**81**	**Ampliseq Ion Torrent**
**447**	**69**	**Ampliseq Ion Torrent**
**447**	**52**	**Ampliseq Ion Torrent**
**447**	**67**	**Ampliseq Ion Torrent**
**460**	**70**	**Ampliseq Ion Torrent**
**460**	**67**	**Ampliseq Ion Torrent**
**460**	**52**	**Ampliseq Ion Torrent**
**484**	**48**	**Nextera MiSeq**
**484**	**87**	**Ampliseq Ion Torrent**
**484**	**52**	**Ampliseq Ion Torrent**

ND: Did not disclose. ‡ Represents suggestive prodromal case.

Table S2. Primer designs.

Sanger Sequencing Verification †					
**Gene**	**Mutation**	**Forward 5′-3′ Primer**	**Reverse 5′-3′ Primer**	**Annealing Temperature °C**	**Size bp**
KCNJ15	**p.R28C**	**TTCTCCTTTCTTGTG-TACTTCC**	**CAGGGTGAGTTT-GTATCTCC**	**57**	**340**
PASK	**p.P519L**	**ATCCTCAGCTGG-GACTGGAG**	**TGGTCACCCATGTCATTA TC**	**57**	**233**
SON	**p.S1595P**	**GCTGGTACTAG-TCCTGTTGG**	**CTGGTGGAAATTAC-CATGTC**	**57**	**281**
SIPA1L1	**p.R236Q**	**GCAGAATAC-GCCAGCGAAG**	**TGACAGAG-TCTTCTGATCGGTTATC**	**60.5**	**465**
ZNF462	**p.I1523V**	**GAGGATGCAA-GACTGTCCCC**	**CAAAGTGCCGTGTGTG-TACG**	**60.5**	**432**
DUSP19	**p.I111R**	**ATGGGTTAG-TAGGTAAGGGAGG**	**TGCCCTCTTGA-TATGTACGAAGC**	**60.5**	**408**
KCTD1	**p.G134R**	**TCGAGTCGTGAT-TCCAGCC**	**TGATTAG-TGACATAACAA-GAAGTGG**	**60.5**	**360**
TAF1C	**p.R346Q**	**CACTGAGCTT-GTCCTCTAGGC**	**GTGCTCTTCCAGCTCTCG G**	**60.5**	**406**
DARS2	**p.S59L**	**ATTTCCCCTG-TAGCCCTGAC**	**ACGTTTTATTCTTCG-GACTGGTTG**	**60.5**	**320**

EXPH5	**p.T920S**	**TTGGTGTTAGCTCAGAA GCATCC**	**AGCAGTTTTCTCATA-CATGGCAGG**	**60.5**	**385**
FAM71B	**p.I318T**	**GCCATCGAGGTG-GAAGTACC**	**CTAG-TGCGGCTTCTCCATCC**	**60.5**	**349**
CCDC180	**p.R1684C**	**GAGAACTCTTGCCAG-TGCCT**	**TGGTAAAGCTACAG-TGCCCG**	**60.5**	**395**
SLC2A12	**p.S357L**	**GGGGCATGAG-TGAGCTTCTG**	**TGTACAAATCAC-TGGCCAACC**	**60.5**	**401**
DOCK3	**p.R392W**	**GCTGGG-GAACAGAGGGAAAG**	**CCTGACTTGGGATGTGG-GAG**	**60.5**	**532**
TPR	**p.K1038N**	**TTTGTAAGGTCCAC-CAGTTCTT**	**AGGGAATGGA-TATGGAGTTGCA**	**60.5**	**395**
DNAH1	**p.K1792R**	**CCTCAAGCGA-GAAAACCCCA**	**CTGACCTG-GACTCCAAGGTG**	**60.5**	**390**
PCDHGA7	**p.S667G**	**AGAAC-GCCTGGCTGTCATAC**	**CAAGCCACCTTCTGAA-GCCT**	**60.5**	**398**
MYOT	**p.N30K**	**GGAGA-CACATCACCCTTTGTCA**	**CCCAGCAGAT-TATTCTTACCACC**	**60.5**	**658**
KIF9	**p.R287W**	**GTGAATCCATGCCAC-CAGGA**	**GCTGACCTGAC-CACAGACTT**	**60.5**	**374**
DNAJC12	**p.T99M**	**ACAGGCTCTTCTGG-CACAAA**	**CTCCGAGAGA-GAAGTGCTCA**	**60.5**	**386**
SLAMF8	**p.V234E**	**TCCTGCATT-GTCTCCAACCC**	**GAGAGGTAGGAAGGGG-CAGA**	**60.5**	**378**
ZNF75A	**p.Q212E**	**GGAAACAA-GAGCTGCTCAAACT**	**CTCAC-CTGTGTGGGTTCTCC**	**60.5**	**392**
**TaqMan Genotyping**					
**Gene**	**Mutation**	**Forward 5′-3′ Primer**	**Reverse 5′-3′ Primer**	**Annealing Tem-perature °C**	**Size**
KCNJ15	**p.R28C**	**CCCCTGGTGAA-GCACACT**	**GCTGTGCCCACTCTT-GGA**	**62**	**75**
		**MGB-Probe(WT): ACAGACCCCGCGTCAT**	**MGB-Probe(MT): AACAGACCCTGCGTCAT**		
**High Resolution Melt Genotyping**					
ARL14EP	**p.A146V**	**TTGTCTGTTGTCAAGAT-TTGAGTTG**	**AGTTTGCCTTCCTGGAT-TCG**	**60**	**105**
SIPA1L1	**p.R236Q**	**AAACAGGGAACATCTG-GAGAAAGC**	**TGCCACCACCAG-TAATGAGAAAG**	**60**	**118**

† Requirements for Sanger sequencing validation was lesser in kindreds that were sequenced across multiple platforms due to likelihood of same machine-artefacts. Therefore, putative variants from families 484 and 431 did not undergo additional Sanger sequencing confirmation.

Table S3. Genotyping variants from #002 by country.

Country	Platform	Cases	Controls
**Australia**	**TaqMan**	**1372**	**1397**
**Canada**	**MassARRAY**	**276**	**70**
**Germany**	**MassARRAY**	**112**	**60**
**Italy**	**TaqMan & MassARRAY**	**1243**	**183**
**Norway**	**MassARRAY**	**1379**	**1095**

**Saskatoon**	**MassARRAY**	**640**	**366**
**South Africa**	**MassARRAY**	**117**	**272**
**Sweden**	**MassARRAY**	**416**	**792**
**Taiwan**	**MassARRAY**	**637**	**320**

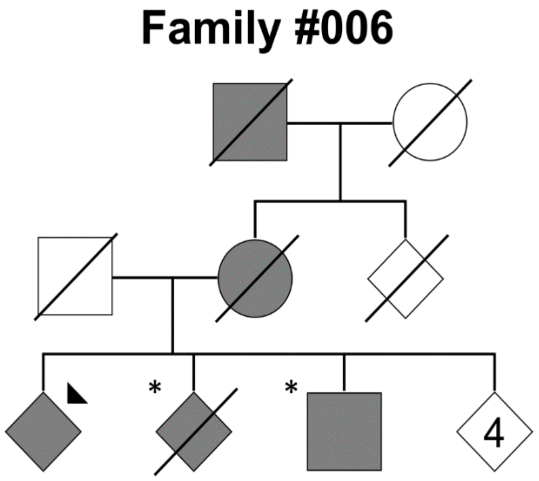

Figure S1. Queensland kindred, #006, with two affected members carrying the *ZNF75A* p.Q212E mutation, as indicated by the star. Whole exome sequencing performed on three affected siblings, finding 20 shared rare sequence variants. Full shaded shapes represent PD. Triangle suggests proband case. Gender disclosed and minimum data shown to protect the privacy of the participants.

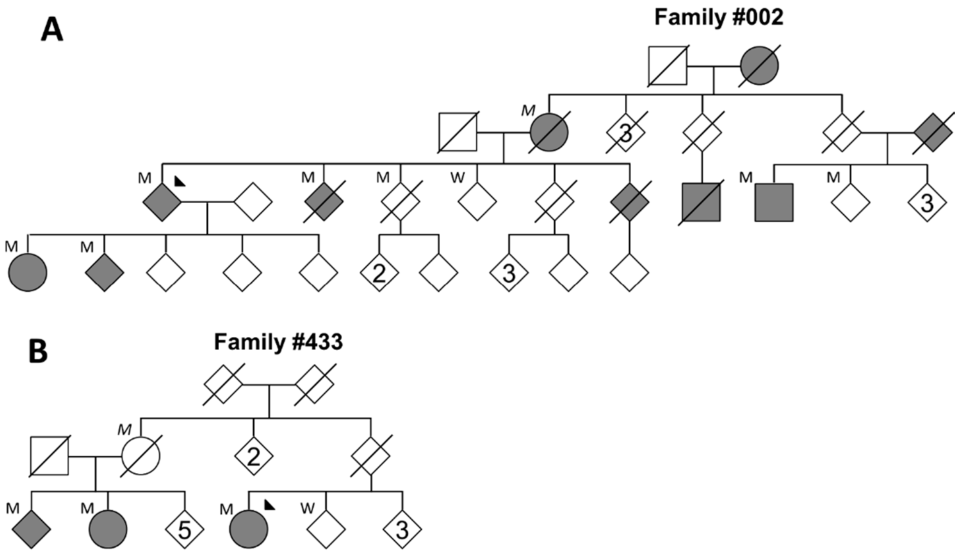

Figure S2. (A) Segregation of the KCNJ15 p.R28C variant in family #002. **(B)** Segregation of SIPA1L1 p.R236Q variant in family #433. Full shaded shapes represent PD. M represents heterozygous mutation carrier. Italicized *M* represents inferred mutation carrier >80 years. W represents confirmed homozygous reference >80 years. Triangle suggests proband case. Gender disclosed and minimum data shown to protect the privacy of the participants.
